# Impact of Apolipoprotein(a) Isoform Size on Lipoprotein(a) Lowering in the HPS2-THRIVE Study

**DOI:** 10.1161/CIRCGEN.117.001696

**Published:** 2018-02-15

**Authors:** Sarah Parish, Jemma C. Hopewell, Michael R. Hill, Santica Marcovina, Elsa Valdes-Marquez, Richard Haynes, Alison Offer, Terje R. Pedersen, Colin Baigent, Rory Collins, Martin Landray, Jane Armitage

**Affiliations:** From the Medical Research Council Population Health Research Unit (S.P., M.R.H., R.H., C.B., J.A.); and the Clinical Trial Service Unit and Epidemiological Studies Unit (S.P., J.C.H., M.R.H., E.V.-M., R.H., A.O., C.B., R.C., M.L., J.A.), Nuffield Department of Population Health, University of Oxford, United Kingdom; Northwest Lipid Metabolism and Diabetes Research Laboratories, University of Washington, Seattle (S.M.); and Center for Preventive Medicine, University of Oslo, Norway (T.R.P.). A complete list of collaborators in HPS2-THRIVE (Heart Protection Study 2–Treatment of HDL to Reduce the Incidence of Vascular Events) is given in reference 13.

**Keywords:** apolipoprotein(a), clinical trial, coronary disease, isoform size, lipoprotein(a), therapeutics

## Abstract

**Background:**

Genetic studies have shown lipoprotein(a) (Lp[a]) to be an important causal risk factor for coronary disease. Apolipoprotein(a) isoform size is the chief determinant of Lp(a) levels, but its impact on the benefits of therapies that lower Lp(a) remains unclear.

**Methods:**

HPS2-THRIVE (Heart Protection Study 2–Treatment of HDL to Reduce the Incidence of Vascular Events) is a randomized trial of niacin–laropiprant versus placebo on a background of simvastatin therapy. Plasma Lp(a) levels at baseline and 1 year post-randomization were measured in 3978 participants from the United Kingdom and China. Apolipoprotein(a) isoform size, estimated by the number of kringle IV domains, was measured by agarose gel electrophoresis and the predominantly expressed isoform identified.

**Results:**

Allocation to niacin–laropiprant reduced mean Lp(a) by 12 (SE, 1) nmol/L overall and 34 (6) nmol/L in the top quintile by baseline Lp(a) level (Lp[a] ≥128 nmol/L). The mean proportional reduction in Lp(a) with niacin–laropiprant was 31% but varied strongly with predominant apolipoprotein(a) isoform size (*P*_Trend_=4×10^−29^) and was only 18% in the quintile with the highest baseline Lp(a) level and low isoform size. Estimates from genetic studies suggest that these Lp(a) reductions during the short term of the trial might yield proportional reductions in coronary risk of ≈2% overall and 6% in the top quintile by Lp(a) levels.

**Conclusions:**

Proportional reductions in Lp(a) were dependent on apolipoprotein(a) isoform size. Taking this into account, the likely benefits of niacin–laropiprant on coronary risk through Lp(a) lowering are small. Novel therapies that reduce high Lp(a) levels by at least 80 nmol/L (≈40%) may be needed to produce worthwhile benefits in people at the highest risk because of Lp(a).

**Clinical Trial Registration:**

URL: https://clinicaltrials.gov. Unique identifier: NCT00461630.

## Abstract 2

Supplemental Digital Content is available in the text.

**See Editorial by Boffa and Koschinsky**

**Clinical Perspective**

Lipoprotein(a) (Lp[a]) is an important causal risk factor for coronary disease, particularly in the fifth of the population with highest Lp(a) levels. The percentage and absolute reductions in Lp(a) with niacin–laropiprant vary strongly with apolipoprotein(a) isoform size and Lp(a) level. In this study, niacin–laropiprant lowered Lp(a) levels by 1 to 34 nmol/L in the bottom to the top fifth of baseline Lp(a) levels, but even in individuals with extreme Lp(a) levels or small apolipoprotein(a) isoform size, the estimated benefits on coronary risk of Lp(a) lowering with niacin–laropiprant are small and, therefore, do not outweigh the adverse effects of niacin. Dependency of Lp(a) reductions on isoform size may apply to Lp(a) reductions with some other therapies as well and affect assessment of the efficacy of the therapy for Lp(a) lowering.

Lipoprotein(a) (Lp[a]) is formed by a low-density lipoprotein (LDL) particle characterized by the presence of apolipoprotein(a) (apo[a])—a protein with a genetically determined variable number of kringle IV (KIV) domains that define apo(a) isoform size. Lp(a) levels are strongly inversely associated with the number of KIV domains and vary widely within, and between, different ethnic groups.^1^ Individuals may carry Lp(a) of 2 distinct apo(a) isoform sizes (derived from the 2 copies of chromosome 6), but because of the inverse relationship between Lp(a) levels and KIV domains, the Lp(a) with the smaller number of KIV domains will tend to be predominantly expressed.

Large studies of 2 genetic variants that are associated with lifelong higher Lp(a) levels have provided evidence of a causal association between Lp(a) levels and cardiovascular disease:^2^ the variant alleles, carried by 1 in 6 people of European ancestry, are associated with ≈50% higher risk of coronary heart disease^3,4^ and, from limited indirect evidence, an Lp(a) difference per variant of 90 to 150 nmol/L.^3,5,6^ A similar strength log-linear relationship between Lp(a) and cardiovascular events risk (equating to a hazard ratio of 1.5 per 80–125 nmol/L) was seen in a recent observational study.^7^ The J-shaped relationship between log coronary heart disease risk and log Lp(a) observed in larger scale evidence from the Emerging Risk Factors Collaboration meta-analysis of observational studies conducted during a wide time span would also be approximately consistent with a log-linear relationship of risk with Lp(a).^8^ However, because mean Lp(a) levels varied >5-fold across the included studies (probably through lack of standardization and assay dependences on apo[a] isoform size in studies pre-dating the availability of reference materials and methods for Lp[a]), this study may not yield a robust estimate of the risk per unit difference in Lp(a).^6,9,10^

The strong genetic determination and highly skewed distribution of Lp(a) are in marked contrast to the features of LDL particles not associated with Lp(a). However, both Lp(a) and other LDL particles are causally associated with coronary disease and are reduced by some of the same lipid-modifying therapies. The most widely used treatment that lowers Lp(a) substantially is nicotinic acid (niacin), which is reported to lower Lp(a) by 30% to 40% on average.^1^ Niacin also lowers LDL cholesterol (LDL-C) and raises high-density lipoprotein cholesterol and has been widely used in the United States as an addition to statin therapy with the intention of producing greater reductions in cardiovascular risk^1,11^ and is under consideration for the prevention of aortic valve disease.^12^ Recent randomized evidence has, however, demonstrated several hazards associated with niacin and, in the HPS2-THRIVE (Heart Protection Study 2–Treatment of HDL to Reduce the Incidence of Vascular Events) and AIM-HIGH (Atherothrombosis Intervention in Metabolic Syndrome with Low HDL/High Triglycerides: Impact on Global Health Outcomes) trials, there were no significant benefits of the extended release niacin formulations on vascular outcomes.^13–15^ The CETP (cholesteryl ester transfer protein) inhibitor, anacetrapib, and PCSK9 (proprotein convertase subtilisin/kexin type 9) inhibitors also lower Lp(a) to a similar extent,^16–20^ in conjunction with lowering LDL-C and other lipid changes.

Absolute reductions in Lp(a) with any of these therapies are likely to vary considerably between individuals with different Lp(a) levels. Proportional reductions may also vary with baseline Lp(a) level (as seen in the LAPLACE (LDL-C Assessment with PCSK9 Monoclonal Antibody Inhibition Combined With Statin Therapy) trial of PCSK9 inhibition where the percentage reduction with evolocumab varied from ≈40%–<20% in the lowest-to-highest quartiles by Lp[a] level^17^) or with the number of KIV domains. More detailed information on factors that influence the magnitude of reductions in Lp(a) is thus needed to assess the potential benefits of such therapies through Lp(a) lowering.

This report considers the Lp(a)- and LDL-lowering effects of extended release niacin plus laropiprant (niacin–laropiprant) in the HPS2-THRIVE randomized trial. It aims to evaluate the reduction in Lp(a) by baseline Lp(a) levels and numbers of KIV domains, to estimate by how much Lp(a) reduction with niacin–laropiprant might be expected to reduce coronary heart disease risk.

## Materials and Methods

The data, analytic methods, and study materials will not be made directly available to other researchers for purposes of reproducing the results or replicating the procedure, but procedures for requesting data access are available on https://www.ndph.ox.ac.uk/about/data-access-policy.

### HPS2-THRIVE Study

Men and women aged 50 to 80 years were recruited in 245 sites in the United Kingdom (UK), Scandinavia, and China and were eligible if they had a history of myocardial infarction, cerebrovascular disease (ischemic stroke or transient ischemic attack), peripheral arterial disease (intermittent claudication or noncoronary arterial surgery or angioplasty), or diabetes mellitus with other evidence of symptomatic coronary disease. There were no lipid entry criteria. The study protocol (available with the primary publication^13^) was approved by the relevant institutional review board for each participating center. Eligible patients were asked to provide written informed consent and to stop any statin therapy. Before randomization, each participant received simvastatin, 40 mg daily; if this dose was not as effective as their prior statin treatment or their total cholesterol was ≥3.5 mmol/L (135 mg/dL) after 4 weeks on simvastatin alone, ezetimibe, 10 mg daily, was added. After this LDL-lowering therapy had been standardized, a baseline blood sample was taken before participants received niacin–laropiprant 1 g/20 mg daily for 4 weeks followed by 2 g/40 mg daily for 3 to 6 weeks. Participants who tolerated this treatment and remained eligible^21^ were randomly allocated to niacin–laropiprant 2 g/40 mg daily or to matching placebo for a median duration of ≈4 years, with the primary outcome results reported in 2014.^13^ Additional details are provided elsewhere.^13,21^ LDL-C was measured in both baseline samples and in samples taken at a median of 1 year post-randomization (interquartile range, 0.94–1.05 years) in 24 205 participants.

### HPS2-THRIVE Lp(a) Substudy

Determination of Lp(a) concentration and apo(a) isoform size was performed in a subsample of 3978 participants from the UK (of white ethnicity) and China who had baseline and follow-up samples from the median 1-year visit. The subsample was balanced for treatment arm and region (Europe/China) but otherwise random. The European component of HPS2-THRIVE was represented by the UK because there were constraints on the use of Scandinavian samples. Only 1% of European participants in HPS2-THRIVE were of nonwhite ethnicity, and samples taken at a median of 1 year post-randomization were available for 96% of surviving participants.

### Laboratory Methods

The procedures for the collection and storage (in liquid nitrogen) of blood samples have been reported previously.^13,21^ The effects of niacin–laropiprant on Lp(a) and LDL-C were to be assessed by comparison of 1-year postrandomization levels in the active and placebo arm (ie, intention-to-treat analyses) within subgroups determined by apo(a) KIV domains and by baseline lipid levels. Determination of plasma Lp(a) levels and number of apo(a) KIV domains was conducted between 2012 and 2014.

In the 1-year samples, Lp(a) was measured in nmol/L at the Northwest Lipid Metabolism and Diabetes Research Laboratories by the double monoclonal antibody-based ELISA reference method.^22^ Apo(a) isoform sizes, defined by the relative numbers of KIV domains, were determined at the same laboratory by high-resolution sodium dodecyl sulfate-agarose gel electrophoresis followed by immunoblotting.^23^ This yielded ≤2 numbers of KIV domains per sample (depending on whether the individual was heterozygous or homozygous) and identified the predominantly expressed isoform. Particles with the lower number of KIV domains were predominant in 79% of participants; hence, in the 11% where the 2 isoforms were equally expressed, the lower number of KIV domains was used as the predominant KIV domains in statistical analyses. In baseline samples, Lp(a) was measured in nmol/L at the central laboratory (Wolfson Laboratories, Clinical Trial Service Unit, Oxford) by a polyclonal antibody-based turbidimetric assay (Denka Seiken) certified by the Lp(a) reference laboratory (Northwest Lipid Metabolism and Diabetes Research Laboratories) to produce results aligned to the ELISA reference method and largely independent of apo(a) isoform size (Methods I and II in the Data Supplement; Figure I in the Data Supplement, which shows that in 2641 samples assayed by both methods, the Spearman correlation coefficient was 0.99). It should be noted that the statistical analyses do not require assumption that the 2 assay methods were completely interchangeable (Statistical Methods). No results were outside the range of the monoclonal antibody assay; by contrast, for Lp(a) at baseline by the polyclonal antibody assay, 286 results were below and 2 were above the assay sensitivity and linearity limits. Assay values outside these limits were available for use in imputation. LDL-C at baseline and follow-up was measured using standard spectrophotometric enzymatic methods at the central laboratory. Assay details of other biochemistry measures considered as potential explanatory variables are provided elsewhere.^21^

### Statistical Methods

Patients were categorized by quintiles of their Lp(a) and LDL-C measurements at baseline (ie, while taking their background study statin-based LDL-lowering therapy but not their randomly allocated niacin–laropiprant or placebo treatment) and by quintiles of their predominant KIV domain number. Where Lp(a) at baseline was outside the analyzable range, it was imputed (Methods III in the Data Supplement). Lp(a) had an approximately log-normal distribution. Thus, analyses of percentage reductions were based on linear regressions of log_e_ Lp(a) levels at 1 year (from the monoclonal assay) as the dependent variable, with adjustment terms for baseline Lp(a) parameters included as covariates to allow for chance differences between the arms and to remove sources of variation and thereby render the analyses more sensitive to identifying the effects of niacin–laropiprant. Baseline Lp(a) parameters included in the regression model were log_e_ baseline Lp(a) (polyclonal assay), quintile of baseline Lp(a) (as a categorical variable, to allow for nonlinear effects), predominant KIV domain number, quintile of predominant KIV domain number (as a categorical variable), region (UK and China), and interactions between region and the 4 Lp(a) and KIV terms. In addition, parallel sensitivity analyses were run using 1-year Lp(a) levels measured by the polyclonal assay, which were available in a subset of 2641 of the 3978 participants (Methods II in the Data Supplement).

Percentage reduction in Lp(a) was estimated as 100×(1−exp[β]), where β denotes the estimated effect of niacin–laropiprant on log_e_ Lp(a). Absolute reductions were estimated with adjustment for strata of quintile of baseline Lp(a) level within region, with bootstrapping (using 10 000 replications) to estimate SEs and confidence intervals. A parallel approach was used for analyses of LDL-C, with adjustment for quintile of baseline LDL-C and log_e_ baseline LDL-C. Bootstrapping was not used for LDL-C because its distribution was adequately normal, given the sample size. Analyses were performed using SAS (version 9.2).

## Results

Baseline characteristics of the 3978 participants in this substudy (Table 1) were similar to those previously reported in the HPS2-THRIVE study overall.^13,21^ Baseline LDL-C and Lp(a) levels were higher in the UK than in China, and there was a marked difference in the KIV domains distribution between the 2 regions, with 27% of UK versus 7% of Chinese participants having KIV domains in the lowest quintile (KIV, ≤17).

**Table 1.**
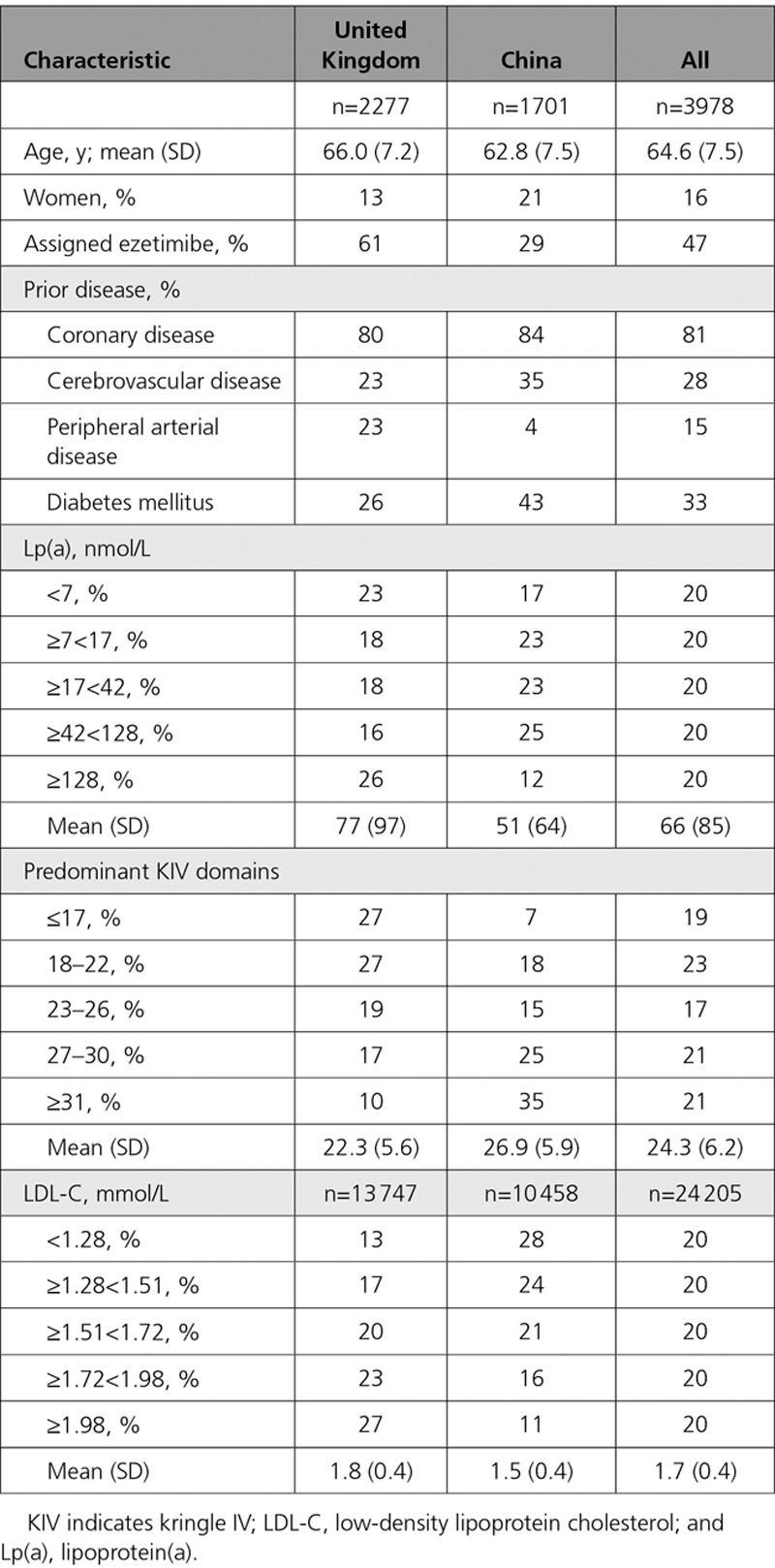
Baseline Characteristics of Participants With Median 1 Year Samples

**Figure 1.**
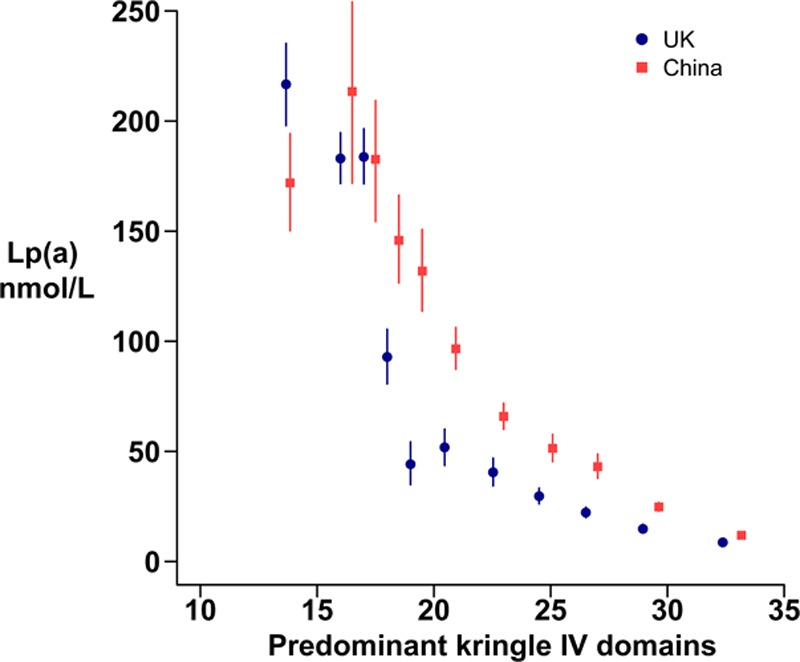
**Baseline lipoprotein(a) (Lp[a]**) **levels by the kringle IV domains of the predominantly expressed apolipoprotein(a**) **isoform, within the United Kingdom and China**. Blue circles denote United Kingdom, and red squares denote China. The 11 kringle IV domain groups shown are ≤15, 16, 17, 18, 19, 20 to 21, 22 to 23, 24 to 25, 26 to 27, 28 to 30, and ≥31. Lp(a) indicates lipoprotein(a).

As expected, there were strong inverse relationships between Lp(a) levels and the KIV domains of the predominant apo(a) isoform in both regions (Figure 1). For a given number of KIV domains, Lp(a) levels tended to be higher in Chinese than in UK participants. The KIV domains of the nonpredominant apo(a) isoform accounted for a much smaller amount of the Lp(a) (Methods IV in the Data Supplement, Figure II in the Data Supplement). Lp(a) levels at baseline and 1 year in the placebo arm were highly correlated (coefficients: Pearson for log_e_ Lp[a], 0.95; Spearman, 0.97), and the baseline Lp(a) adjustment terms for the analysis of the percentage Lp(a) reduction (defined in the Methods) explained 92% of the variance of the 1-year Lp(a) levels in the placebo arm. The Pearson correlation coefficient between LDL-C levels at baseline and 1 year in the placebo arm was 0.58. There was little difference in mean baseline LDL-C between the 2 randomized arms (<0.01 mmol/L, niacin–laropiprant minus placebo) because the randomized allocation was balanced for LDL-C (as well as for several other factors but not for Lp[a]).^13^ By chance, in the subset of the trial population with Lp(a) measured, the unadjusted mean baseline Lp(a) was 3.7 (SE, 2.7) nmol/L higher in the niacin–laropiprant arm than in the placebo arm.

### Reductions in Lp(a) With Niacin–Laropiprant

**Figure 2.**
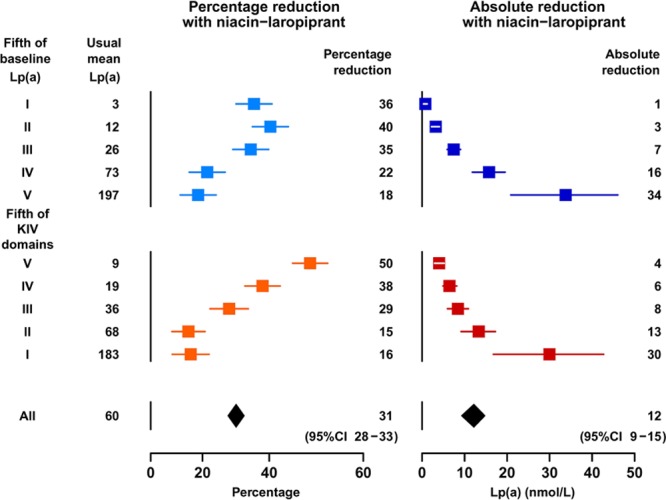
**Percentage and absolute reductions in lipoprotein(a) (Lp[a]) with niacin–laropiprant by quintiles of baseline Lp(a) levels and kringle IV domains**. Percentage reduction panels show adjusted Lp(a) reductions based on modeling log_e_ Lp(a) (Methods) and are plotted with an x scale linear in the log_e_ Lp(a) difference. Absolute reductions (with adjustment by stratification) are plotted on linear scales. Usual mean Lp(a) is the mean Lp(a) in the placebo arm at 1 y.

At the median 1-year visit, compliance with taking study niacin–laropiprant was 86% among participants in this substudy allocated active treatment. Figure 2 shows Lp(a) reductions with niacin–laropiprant at 1 year within quintiles by baseline Lp(a) level and quintiles by KIV domains (shown in reverse order) among the 3978 participants with Lp(a) measured at both time points (additional details of the estimation are given in Table I in the Data Supplement). The percentage reduction in Lp(a) was 31% (95% confidence interval, 28%–33%) overall but attenuated from 36% to 18% across quintiles by increasing baseline Lp(a) (*P*_Trend_=2×10^−8^) and attenuated even more strongly across quintiles by decreasing KIV domains, varying from 50% in the highest KIV quintile to 16% in the lowest KIV quintile (*P*_Trend_=4×10^−29^). Consistent with this, in China, where KIV domains tended to be higher than in the UK (Table 1), the percentage reduction in Lp(a) with niacin–laropiprant was also higher than in the UK (38% versus 25%; *P*_Diff_=2×10^−5^).

The overall absolute reduction in Lp(a) was 12.2 (95% confidence interval, 9.3–14.8) nmol/L (20% of the overall adjusted mean level in the placebo arm of 60 nmol/L: Figure 2) but increased with baseline Lp(a) level in a less than pro rata manner, such that in the top Lp(a) quintile, where the mean Lp(a) was ≈200 nmol/L, the reduction was 33.8 (95% confidence interval, 20.9–46.1) nmol/L.

In the quintile with the lowest Lp(a) levels, Lp(a) may be subject to more measurement error, and Lp(a) lowering would be least relevant. When this quintile was excluded, the trends in the percentage reductions in Lp(a) with niacin–laropiprant across Lp(a) quintiles and across KIV quintiles were even stronger (*P*_Trend_=2×10^−13^ versus 2×10^−8^ across Lp[a] quintiles and *P*_Trend_=2×10^−32^ versus 4×10^−29^ across KIV quintiles; Table II in the Data Supplement), and the average percentage reduction in Lp(a) fell slightly (from 31% to 30%).

Further analyses (Table 2) showed that the trend in the percentage reductions in Lp(a) with KIV domains accounted for almost all of the trend in the percentage reductions in Lp(a) with baseline Lp(a) level and region (*P* for residual association, 0.01 and 0.30, respectively). In marked contrast to the highly significant association with KIV domains, no other baseline characteristics showed statistically significant (allowing for multiple testing of 15 factors) independent influence on the percentage reductions in Lp(a) with niacin–laropiprant (all *P*>0.005 [Bonferroni corrected *P*>0.05]; Table III in the Data Supplement). It is noteworthy that the number of KIV domains in the nonpredominant isoform did not show any independent influence on the Lp(a) reduction.

**Table 2.**
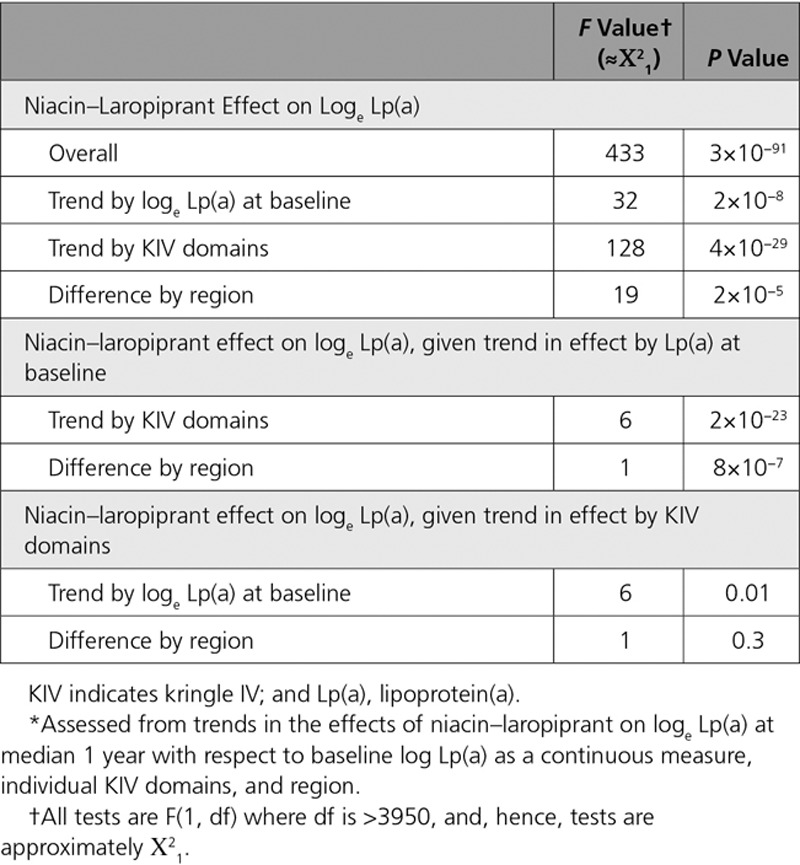
Comparison of the Relative Strengths of the Trends in the Percentage Reductions in Lp(a) With Niacin–Laropiprant by Baseline Lp(a), KIV Domains, and region*

In contrast to the varying percentage reductions in Lp(a) by baseline Lp(a) level, there was ≈20% reduction in LDL-C with niacin–laropiprant irrespective of baseline LDL-C level (in analyses based on 24 205 participants with LDL-C measured at both baseline and at a median of 1 year). Correspondingly, the absolute reductions increased from 0.21 to 0.40 mmol/L in proportion to the baseline level (Figure III in the Data Supplement).

### Sensitivity Analyses

Parallel analyses of the effect of niacin–laropiprant on reductions in Lp(a) measured in the 1-year samples by the monoclonal and polyclonal assays were conducted in the subset of 2641 participants with Lp(a) determined by both methods. The 2 methods gave similar results, with the trends in the percentage reductions by quintiles of KIV domains remaining highly significant (Table II in the Data Supplement). For both assays, the percentage reductions in Lp(a) were markedly higher in Lp(a) quintiles II and III than in quintiles IV and V. However, the polyclonal assay had more limited ability to measure low Lp(a), resulting in the exclusion of 332 participants with results below the limit of the assay. This distorted the observed reductions in the lowest quintile of baseline Lp(a) levels, reduced the overall percentage reduction in Lp(a) in the subset with the polyclonal measurements to ≈24% to 25% (compared with 31% in the whole study) and increased the absolute reduction slightly from 12 to 14 nmol/L, which demonstrates that limitations of assay range for Lp(a) may affect the results.

## Discussion

In this substudy of HPS2-THRIVE, niacin–laropiprant resulted in a mean percentage reduction in Lp(a) of 31%, in line with the previously reported effects of niacin.^1^ However, because Lp(a) reductions depended strongly on apo(a) isoform size, the mean Lp(a) was lowered by 12 nmol/L overall, only 20% of the overall adjusted mean level in the placebo arm of 60 nmol/L. In participants in the top quintile by Lp(a) levels, the mean percentage and absolute reductions were 18% and 34 nmol/L, respectively (Figure 2).

Large-scale genetic studies provide a basis for estimating the strength of the causal association between Lp(a) levels and coronary risk following the Mendelian randomization principle. Such studies have found an odds ratio of ≈1.5 for coronary risk associated with carrying particular variants in the *LPA* gene,^3,4^ and indirect published evidence supplemented by direct unpublished evidence using assays well aligned to the reference method (Table IV in the Data Supplement) suggests an Lp(a) difference of ≈110 to 115 nmol/L per variant. Mendelian randomization studies of LDL-C have found an odds ratio for coronary disease of ≈2 per 1 mmol/L higher LDL-C, which is much stronger than the effect seen in statin trials^24^ and so suggest that only a third to a half of the coronary risk per unit LDL-C from lifelong genetically elevated LDL-C is reversed within a few years of statin therapy.^24,25^ Assuming the same holds for Lp(a) then, taken together, the literature suggests that therapeutic lowering of Lp(a) during a trial might be expected to produce a 15% to 20% reduction in coronary risk per 100 nmol/L lower Lp(a). Similar sized benefits of Lp(a) lowering may be seen for peripheral vascular disease outcomes, but the potential benefits for stroke appear to be smaller.^26,27^

The Lp(a) reductions with niacin of 12 nmol/L overall and 34 nmol/L in the top quintile by Lp(a) levels in the present study might, therefore, have been expected to produce reductions in the risk of coronary events of only ≈2% and 5% to 6%, respectively. Hence, compared with the contribution from the LDL-C reduction of 0.3 mmol/L with niacin–laropiprant in the HPS2-THRIVE trial, which would be expected to reduce major vascular events risk by 5% to 6% (Methods V in the Data Supplement),^13^ the overall benefits of the Lp(a) lowering achieved with niacin–laropiprant are likely to be much smaller.

No significant reduction in cardiovascular events with niacin was found in either the HPS2-THRIVE or AIM-HIGH trials, but there were significant excesses in the rates of various serious adverse events (including those related to diabetes mellitus, gastrointestinal, musculoskeletal, skin, infectious, and bleeding outcomes) among the niacin-allocated participants.^13–15^ There was no trend in the excess of these serious adverse events with Lp(a) level in the present substudy (data not shown). Therefore, even in participants with high Lp(a), any benefits of niacin do not seem to outweigh the hazards.

The present results indicate that the effects of Lp(a)-lowering therapies should typically be considered in terms of their absolute reductions in Lp(a) levels rather than in terms of their proportional effects^28^ because the proportional reductions can vary by baseline Lp(a) level (as well as be unduly influenced by the extent to which an assay is able to measure low Lp[a] levels; Figure 2; Table II in the Data Supplement). To produce 12% to 15% reductions in risk through Lp(a) lowering, novel therapies that reduce high Lp(a) levels by at least 80 nmol/L (ie, 40% among those in the top quintile of Lp[a] levels where mean Lp[a] was ≈200 nmol/L) are likely to be needed. Emerging therapies under evaluation, such as antisense oligonucleotides targeting apo(a) mRNA, hold the potential to achieve such reductions.^29^

### Impact of KIV Domains

The percentage reduction in Lp(a) varied strongly with both baseline Lp(a) level and the number of KIV domains in the predominant apo(a) isoform (Figure 2). The trend with predominant KIV domains (from 16% to 50% across quintiles; *P*=4×10^−29^) was statistically much stronger than the trend with Lp(a) level (from 18% to 36% across quintiles; *P*=2×10^−^^8^) and remained highly statistically significant after adjustment for the trend with Lp(a) level (Table 2). This shows that niacin–laropiprant had a greater impact on larger apo(a) isoform Lp(a) particles. Similar percentage reductions in Lp(a) have been reported with the PCSK9 inhibitor evolocumab: a reduction in Lp(a) of 32% overall with the most effective regimen, and, as for niacin-laropiprant, there was a trend toward smaller percentage reductions at higher Lp(a) levels.^17^ This trend may also be due (at least in part) to a dependency on apo(a) isoform size, but such analyses have not been reported from any PCSK9 inhibitor trial. The CETP inhibitor anacetrapib lowered Lp(a) by 35% to 50% in 2 studies, but reductions by baseline Lp(a) level or apoa(a) isoform size have not been reported.^16,30^

Dependencies of Lp(a) reductions on apo(a) isoform size would not only affect assessment of the efficacy of Lp(a)-lowering therapies but could also provide insight into mechanisms determining Lp(a) levels, which are poorly understood. The wide variation in plasma Lp(a) levels has been attributed to variation in the rates of hepatic production of Lp(a) of different apo(a) isoform size.^31^ Apo(a) glycosylation affecting Lp(a) synthesis,^32^ and kidney function affecting Lp(a) excretion,^33,34^ have also been reported to have apo(a) isoform-dependent effects. Mounting evidence indicates that niacin acts by reducing Lp(a) production through inhibiting apo(a), and possibly Lp(a)-apoB-100, production.^28,35,36^ CETP inhibition has also been reported to reduce Lp(a) by decreasing the production of apo(a).^37^ ApoB antisense and microsomal triglyceride transfer protein inhibitors block the production of Lp(a) and other apoB-containing lipoprotein particles by blocking apoB production.^28^ PCSK9 inhibition and *APOE* ε2/ε3/ε4 genotype have been demonstrated to affect clearance of Lp(a) via the LDL receptor,^38–40^ but the importance of this route of clearance remains debated, and PCSK9 may also act by modifying apoB production.^28,39,41^ Confirmation that the suggested mechanisms of action for particular therapies can explain any observed trends in the therapeutic Lp(a) reductions with Lp(a) levels, and KIV domains would help consolidate the explanations.^17,42^

The lack of much association of the nonpredominant KIV domains with Lp(a) (Figure II in the Data Supplement) is consistent with a previous report.^5^ This observation, and differences in the mean Lp(a) levels for a given KIV domain by region (Figure 1), may reflect further sources of genetic variation and mechanisms beyond a direct relationship with KIV domains. At the *LPA* locus, the allele frequency for the loss-of-function variant rs41272114 (associated with substantially lower Lp[a]) is ≈0.04 in the UK but negligible in China, and so this could account for some of the difference between the regions. Other genetic variants at the *LPA* locus have also been associated with Lp(a) levels independently of KIV domains.^5,43^

The present study has several strengths: it included a large number of participants; it used the gold standard methods for measuring Lp(a) levels at 1 year and for determining the number of KIV domains; it included 2 distinct ethnic groups, improving the ability to distinguish Lp(a) and KIV effects; and it used a powerful statistical analysis (and avoided bias caused by correlated measurement errors that can occur when change in Lp[a] is compared across groups defined by the same baseline measurement used to define change in Lp[a]^44^).

## Summary

Lp(a) is an important risk factor for coronary disease, particularly among individuals with genetic variation resulting in small apo(a) isoform size and high Lp(a) levels. However, even in individuals with extreme Lp(a) levels, the potential benefits of Lp(a) lowering with niacin–laropiprant on coronary event risk are likely to be small and would not be expected to outweigh the adverse effects of niacin.^13,15^ The impact of niacin–laropiprant varied substantially with apo(a) isoform size and, therefore, dependence on apo(a) isoform size or Lp(a) levels should be considered when assessing the potential benefits of novel Lp(a)-lowering therapies. Knowledge about variation in response by apo(a) isoform size may also be valuable for elucidating the mechanisms determining Lp(a) levels.

## Sources of Funding

This work was supported by grants from Merck, the UK Medical Research Council (MRC_MC_U137686853), the British Heart Foundation (CH/1996001/9454), and Cancer Research UK to the University of Oxford for work designed and conducted independently of the funders.

J.C. Hopewell acknowledges support from the British Heart Foundation (FS/14/55/30806) and the British Heart Foundation Centre of Research Excellence, Oxford.

## Disclosures

The Clinical Trial Service Unit authors (S. Parish, J.C. Hopewell, M.R. Hill, E. Valdes-Marquez, R. Haynes, A. Offer, C. Baigent, R. Collins, M. Landray, and J. Armitage) also report grants from Abbott/Solvay/Mylan, AstraZeneca, Bayer Germany, Novartis, Pfizer, the National Institute for Health Research, UK Biobank, and Wellcome Trust to the University of Oxford for work outside the submitted work. R. Collins reports a prize from Pfizer. R. Collins and S. Parish have a patent for a statin-related myopathy genetic test with royalties paid to University of Oxford and the Medical Research Council from Boston Heart Diagnostics (R. Collins and S. Parish have waived any personal reward). The Clinical Trial Service Unit has a staff policy of not accepting honoraria or other payments from the pharmaceutical industry, except for the reimbursement of costs to participate in scientific meetings. S. Marcovina reports personal fees from Denka Seiken outside the submitted work and a patent Methods and Materials for the Immunoassay of Apolipoprotein(a) and Lipoprotein(a) without royalties paid. T.R. Pedersen reports grants and personal fees from Merck, Pfizer, and Amgen, during the conduct of the study.

## Supplementary Material
