# Supplementary material for: Impact of Apolipoprotein(a) Isoform Size on Lipoprotein(a) Lowering in the HPS2-THRIVE Study
Source: Circ Genom Precis Med. 2018 Feb 15;11(2):e001696. doi: 10.1161/CIRCGEN.117.001696 (PMC5841847; doi:10.1161/CIRCGEN.117.001696)
Supplement: Supplementary file 1 [file hcg-11-e001696-s001.pdf]

SUPPLEMENTAL MATERIAL

Supplemental Methods

1. Denka Seiken polyclonal assay for Lp(a) at the central laboratory
2. Comparison of results by the Denka Seiken and ELISA assays
3. Imputation of polyclonal Lp(a) levels above and below the assay limits
4. Estimation of Lp(a) levels associated with predominant and non-predominant KIV domains jointly
5. LDL-C-lowering with niacin-laropiprant

Supplemental References

Supplemental Table 1

Supplemental Table 2

Supplemental Table 3

Supplemental Table 4

Supplemental Figure 1

Supplemental Figure 2

Supplemental Figure 3

Supplemental Methods

1. Denka Seiken polyclonal assay for Lp(a) at the central laboratory

The central laboratory at CTSU used a Denka Seiken turbidimetric polyclonal antibody method for measuring levels of Lp(a). The assay uses five independent calibrators with Lp(a) levels ranging from low to high. Each calibrator level is derived from pooled sera with similar apo(a) isoform size and each level has an accuracy-based target value assigned by the Northwest Lipid Metabolism and Diabetes Research Laboratories (NLMDRL) using the ELISA reference method, effectively minimizing the inaccuracy of results due to the size variation of apo(a). In addition, the method established in the central laboratory was certified by the NLMDRL to provide accurate Lp(a) values traceable to the WHO-IFCC Reference Material SRM-2B. The accuracy of results is verified by analysing in duplicate on two separate days a set of 80 fresh-frozen samples from individual donors provided by the NLMDRL. The samples were collected from apparently healthy individuals and selected to represent a large range of Lp(a) levels and apo(a) isoform size. An accuracy-based target value in nmol/L was assigned to each sample by the ELISA reference method calibrated with the WHO-IFCC Reference material and each value is the mean of at least 40 analyses performed in duplicate over a period of several weeks. Lp(a) values obtained on the 80 samples by the CTSU central laboratory were compared to those assigned by the NLMDRL. The correlation equation between the obtained and the assigned values was as follows: $r = 0.997$, $y = 0.95x + 1.3$. The apo(a) kringle IV domains number accounted for 0.5% of the bias variation and the results met the established strict criteria for assay accuracy and precision.

The Denka Seiken polyclonal assay for Lp(a) at the central laboratory was run an AU680 Beckman-Coulter analyser, which has an advantage for large-scale studies of ability to measure Lp(a) concurrently with other chemistries (e.g., cholesterol fractions, triglycerides and apolipoproteins), with a throughput of 500 samples per day. The assay is being used for ~500K samples in UK Biobank.

2. Comparison of results by the Denka Seiken and ELISA assays

In a subset of 2976 of the 1-year samples in the present study with Lp(a) assayed by the monoclonal antibody-based reference method at NLMDRL, Lp(a) was also assayed by the Denka Seiken polyclonal assay at the central laboratory. Whereas all results were within the analysable range of the monoclonal assay, for the polyclonal assay, 332 results were below the lower limit of the assay and 3 were above the upper limit. Results by the two assays showed a high degree of concordance (Spearman correlation coefficient 0.99 among the 2641 results within the analysable range and likewise 0.99 over all 2976 results; Supplemental Figure 1).

Supplemental Figure 1 shows (for the 1-year samples) that polyclonal results outside the assay range were similar to the monoclonal results, particularly in the upper range, but even among the 332 samples where the polyclonal result was below the assay limit the correlation with the monoclonal assay results was 0.63. Therefore polyclonal assay results outside the analysable range are suitable for use as imputed values in contexts where imputation is appropriate.

3. Imputation of polyclonal Lp(a) levels above and below the assay limits

Among the 3978 participants in the main analyses, Lp(a) at baseline by the polyclonal assay was below and above the assay limits, respectively, in 286 and 2 participants (and reported results ranged from 3.6 to 583 nmol/L). As baseline Lp(a) was to be used for categorisation and as a covariate, imputation was appropriate and measured values outside the assay range were used as the imputed value.

Polyclonal Lp(a) results from the 1-year samples were used in sensitivity analyses to assess reductions in Lp(a) with niacin-laropiprant, and were not imputed when they were outside the analysable range, as imputed values might not be sufficiently accurate for the assessment of reductions. Reported results from the monoclonal Lp(a) assays on the 1-year samples ranged from 0.01 to 709 nmol/L and no results were outside the analysable range.

4. Estimation of Lp(a) levels associated with predominant and non-predominant KIV domains jointly

Lp(a) levels associated with the predominant and non-predominant KIV domains numbers jointly were estimated within each region separately using a linear regression (with no intercept) on categorical variables defining 11 groups of the predominant KIV domains (as in Figure 1) and, as low domain numbers were infrequent among the non-predominant isoforms, 5 groups (≤17, 18-21, 22-26, 27-31, ≥31) of the non-predominant KIV domains:

Lp(a) = Predominant_KIV_group + Non-predominant_KIV_group

This yielded Lp(a) estimates for each predominant KIV domains group directly and for each non-predominant KIV domains group relative to the highest non-predominant KIV domains group. The highest non-predominant KIV domains group was plotted as having zero Lp(a), since an arbitrary positioning was required and Lp(a) levels were very low at high KIV domains.

5. LDL-C-lowering with niacin-laropiprant

Niacin-laropiprant was associated with an absolute reduction of 0.29 mmol/L LDL-C in the 1-year samples, and, in a previous report, a study average reduction of 0.26 mmol/L that was expected (on the basis of meta-analyses of statin trials) to cause a 5-6% reduction in major vascular event risk.[3] Lp(a) contributes to assayed LDL-C,[4] and so a proportion of the apparent LDL-C reduction would be from Lp(a). Assuming that an Lp(a) particle contains a typical amount of cholesterol for an LDL-particle (~2500 molecules),[5-7] then the 12 nmol/L reduction in Lp(a) might constitute about 0.03 mmol/L of the 0.29 mmol/L reduction in assayed LDL-C in the 1-year samples. Hence, the estimates for the reductions in risk from LDL-C and Lp(a) lowering are likely to double-count by only about 0.5%.

Supplemental references

1. Erqou S, Kaptoge S, Perry PL, Di Angelantonio E, Thompson A, White IR, et al. Lipoprotein(a) concentration and the risk of coronary heart disease, stroke, and nonvascular mortality. *JAMA*. 2009; 302: 412-23.

2. Brown WV, Ballantyne CM, Jones PH, Marcovina S. Management of Lp(a). *J Clin Lipidol*. 2010; 4: 240-7.

3. HPS2-THRIVE Collaborative Group. Effects of extended-release niacin with laropiprant in high-risk patients. *N Engl J Med*. 2014; **371**: 203-12.

4. Nauck M, Warnick GR, Rifai N. Methods for measurement of LDL-cholesterol: a critical assessment of direct measurement by homogeneous assays versus calculation. *Clin Chem*. 2002; **48**: 236-54.

5. Lippi G, Guidi G. Lipoprotein(a): from ancestral benefit to modern pathogen? *QJM*. 2000; **93**: 75-84.

6. Parish S, Offer A, Clarke R, Hopewell JC, Hill MR, Otvos JD, et al. Lipids and lipoproteins and risk of different vascular events in the MRC/BHF Heart Protection Study. *Circulation*. 2012; **125**: 2469-78.

7. Cromwell WC, Otvos JD, Keyes MJ, Pencina MJ, Sullivan L, Vasan RS, et al. LDL Particle Number and Risk of Future Cardiovascular Disease in the Framingham Offspring Study - Implications for LDL Management. *J Clin Lipidol*. 2007; **1**: 583-92.

8. Ronald J, Rajagopalan R, Cerrato F, Nord AS, Hatsukami T, Kohler T, et al. Genetic variation in LPAL2, LPA, and PLG predicts plasma lipoprotein(a) level and carotid artery disease risk. *Stroke*. 2011; 42: 2-9.

9. Clarke R, Peden JF, Hopewell JC, Kyriakou T, Goel A, Heath SC, et al. Genetic variants associated with Lp(a) lipoprotein level and coronary disease. *N Engl J Med*. 2009; 361: 2518-28.

10. Hopewell JC, Parish S, Offer A, Link E, Clarke R, Lathrop M, et al. Impact of common genetic variation on response to simvastatin therapy among 18 705 participants in the Heart Protection Study. *Eur Heart J* 2013; 34:982-92.

Supplemental Table 1: Regression estimates for the effect of niacin-laropiprant on $\log_e$ Lp(a) and corresponding estimated percentage reductions in Lp(a), by quintiles of baseline Lp(a) levels and kringle IV domains.

	N	Usual mean Lp(a), nmol/L	Effect of niacin-laropiprant on $\log_e$ Lp(a) (SE)	Corresponding percentage reduction* (95% CI)
**Quintile of baseline Lp(a), nmol/L**				
≤ 7	794	3.0	-0.45 (0.04)	35.9 (30.8-40.7)
7 – 17	797	11.6	-0.52 (0.04)	40.3 (35.5-44.7)
17 – 42	796	26.1	-0.43 (0.04)	35.0 (29.8-39.8)
42 – 128	795	72.9	-0.24 (0.04)	21.6 (15.3-27.4)
≥ 128	796	196.7	-0.20 (0.04)	18.5 (11.9-24.5)
**Quintile of KIV domains**				
≥ 31	817	9.2	-0.69 (0.04)	49.7 (45.8-53.4)
27 – 30	816	19.2	-0.48 (0.04)	38.2 (33.4-42.7)
23 – 26	688	36.2	-0.34 (0.04)	28.7 (22.6-34.3)
18 – 22	918	68.2	-0.16 (0.04)	15.0 (8.7-20.9)
≤ 17	739	183.1	-0.17 (0.04)	15.8 (8.9-22.2)
**All**	3978	60.4	-0.37 (0.02)	30.8 (28.3-33.2)

Percentage reductions in Lp(a) were estimated as 100(1-exp(β)), where β denotes the regression estimate for the effect of niacin-laropiprant on $\log_e$ Lp(a).

Supplemental Table 2: Percentage and absolute reductions in Lp(a) with niacin-laropiprant by quintiles of baseline Lp(a) and kringle IV domains: monoclonal versus polyclonal Lp(a) assay comparison

Quintile	Lp(a) levels in one year samples from niacin-laropiprant compared to placebo allocated participants						
	Monoclonal Lp(a)					Polyclonal Lp(a)	
	All participants, n=3978			Subset with polyclonal, n=2641*		Subset with polyclonal, n=2641*	
	Usual mean Lp(a)	Percentage reduction	Absolute reduction	Percentage reduction	Absolute reduction	Percentage reduction	Absolute reduction
**Overall**	60.4 (1.8)	30.8 (28.3-33.2)	12.2 (9.7-14.7)	24.0 (21.4-26.6)	13.5 (10.5-16.5)	24.6 (22.6-26.6)	14.4 (11.6-17.2)
**Lp(a) quintiles II-V**	75.3 (2.2)	29.5 (27.2-31.7)	15.0 (11.9-18.1)	24.8 (22.1-27.3)	15.1 (11.7-18.4)	25.9 (23.8-27.9)	16.0 (12.8-19.1)
**Quintiles of baseline Lp(a)**							
I	3.0 (0.2)	35.9 (30.8-40.7)	0.8 (-4.7-6.3)	17.7 (8.5-25.9)	0.0 (-9.2-9.2)	13.3 (5.9-20.1)	0.5 (-8.1-9.1)
II	11.6 (0.3)	40.3 (35.5-44.7)	3.2 (-2.3-8.7)	27.4 (21.9-32.5)	2.3 (-4.1-8.7)	26.0 (21.7-30.1)	2.3 (-3.6-8.3)
III	26.1 (0.6)	35.0 (29.8-39.8)	7.4 (2.0-12.9)	32.3 (27.3-36.9)	6.9 (0.8-13.1)	34.6 (30.9-38.1)	7.7 (2.0-13.5)
IV	72.9 (1.6)	21.6 (15.3-27.4)	15.8 (10.3-21.2)	20.1 (14.2-25.5)	14.1 (7.9-20.2)	22.9 (18.5-27.0)	16.0 (10.2-21.8)
V	196.7 (4.5)	18.5 (11.9-24.5)	33.8 (28.3-39.2)	18.2 (12.0-23.9)	37.7 (31.3-44.1)	18.7 (13.9-23.1)	38.5 (32.5-44.5)
P-values for trend:							
I-V		2×10^{-8}		0.2		0.4	
II-V		2×10^{-13}		2×10^{-3}		4×10^{-4}	
**Quintiles of baseline KIV**							
V	9.2 (0.8)	49.7 (45.8-53.4)	4.0 (-1.2-9.2)	35.0 (29.6-40.0)	4.0 (-2.7-10.8)	30.2 (25.8-34.5)	3.8 (-2.7-10.2)
IV	19.2 (0.8)	38.2 (33.4-42.7)	6.5 (1.2-11.7)	32.3 (26.9-37.3)	6.2 (-0.2-12.7)	32.6 (28.5-36.5)	7.1 (0.9-13.2)
III	36.2 (1.7)	28.7 (22.6-34.3)	8.4 (2.7-14.1)	26.3 (20.1-32.1)	9.1 (2.2-16.0)	28.0 (23.3-32.5)	10.4 (3.8-16.9)
II	68.2 (2.9)	15.0 (8.7-20.9)	13.3 (8.4-18.3)	14.1 (8.0-19.7)	14.5 (8.7-20.2)	18.1 (13.6-22.3)	17.3 (11.9-22.8)
I	183.1 (5.4)	15.8 (8.9-22.2)	30.0 (24.5-35.4)	14.3 (7.7-20.5)	32.4 (26.2-38.7)	15.8 (10.8-20.6)	31.7 (25.7-37.6)
P-values for trend:							
I-V		4×10^{-29}		1×10^{-11}		5×10^{-10}	
I-V (excluding Lp(a) quintile I)		2×10^{-32}		4×10^{-12}		6×10^{-13}	

* Excludes 332 below and 3 above the analysable range for the polyclonal assay.

Supplemental Table 3: The influence of further baseline characteristics on the proportional reduction in Lp(a) with niacin-laropiprant, after allowance for the influence of KIV domains

Baseline characteristic	F-value* (~ X^2_1)	P-value	+/-†
Age	1.16	0.28	-
Female	4.59	0.03	+
Ezetimibe assigned	0.46	0.50	+
Body mass index	0.32	0.57	-
Body surface area	4.81	0.03	-
Coronary heart disease	0.59	0.44	-
Cerebrovascular disease	0.13	0.71	-
Peripheral arterial disease	0.03	0.86	+
Diabetes	1.18	0.28	-
eGFR	3.56	0.06	+
LDL-C	6.67	0.01	+
HDL-C	3.09	0.08	+
Log_e triglycerides	0.56	0.45	-
Apolipoprotein B	4.55	0.03	+
Non-predominant KIV domains	0.02	0.88	+

* All tests are F(1, df2) where df2 is >3950, and, hence, tests are approximately X^2_1
† +(-) Indicates a greater proportional reduction in Lp(a) with higher (lower) levels of the characteristic
KIV = kringle IV;

Supplemental Table 4: Per *LPA* variant Lp(a) differences in the HPS study and in the literature

				Source information*			Estimations (as necessary)*			
Source	Assay	*LPA* variants	N with variant/ total	Overall mean $\log_e$ Lp(a)	Per variant difference in $\log_e$ Lp(a)	Per variant difference in Lp(a)	Log_e Lp(a) with 0 variants†	Log_e Lp(a) with 1 variant	Difference in geometric mean Lp(a) for 1 vs 0 variants	Lp(a) per variant, nmol/L
**Indirect estimation**										
Ronald[8] Cases plus controls	Mono, nmol/L	rs10455872 (af=0.08)	~230/1496	3.3	1.7		3.2	4.9	110	116‡
Ronald[8] Cases plus controls	Mono, nmol/L	rs3798220 (af=0.02)	~59/1496	3.3	1.7		3.0	4.7	90	96‡
Clarke[9] Figure 3	Poly, mg/dl	rs10455872 or rs3798220				~ 50 mg/dl				100-150§
**Direct estimation**										
HPS‖	Poly, nmol/L aligne d to ref assay	rs10455872	256/500			111 (SE 5)				111 (SE 5)
		rs3798220	51/500			133 (SE 9)				133 (SE 9)
		rs10455872 or rs3798220	300/500		1.8	115 (SE 5) nmol/L	2.7	4.8	109	115 (SE 5)

* Direct information available and estimations from indirect source, as available

† L – 2D af, where af is the variant allele frequency, D is the difference in $\log_e$ Lp(a) per variant and L is the overall mean $\log_e$ Lp(a).

‡ Assuming that the difference in the geometric means underestimates the per variant difference in Lp(a) by the same amount as in HPS (6 nmol/L), as this is an attribute of the approximately log-normal distribution of Lp(a).

§ Conversion factor from mg/dl to nmol/L has apo(a) isoform size dependency and varies from about 2-3.[2]

‖ Unpublished data from the Heart Protection Study (HPS) in a random sample of previously genotyped participants with and without the *LPA* variants, rs10455872 and rs3798220[10]; Lp(a) was assayed by the Denka Seiken polyclonal assay, as used in HPS2-THRIVE, in samples taken off statin.

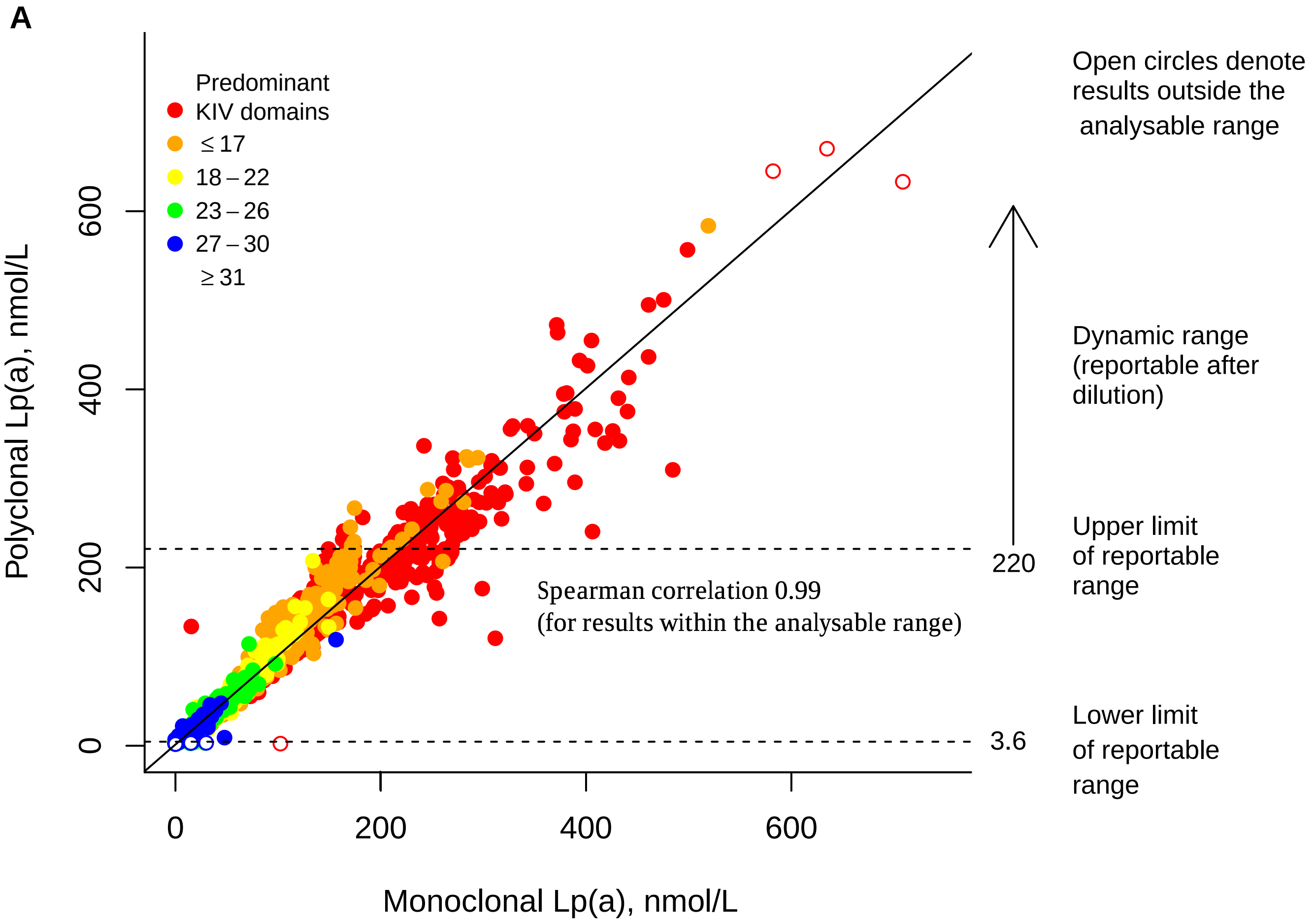

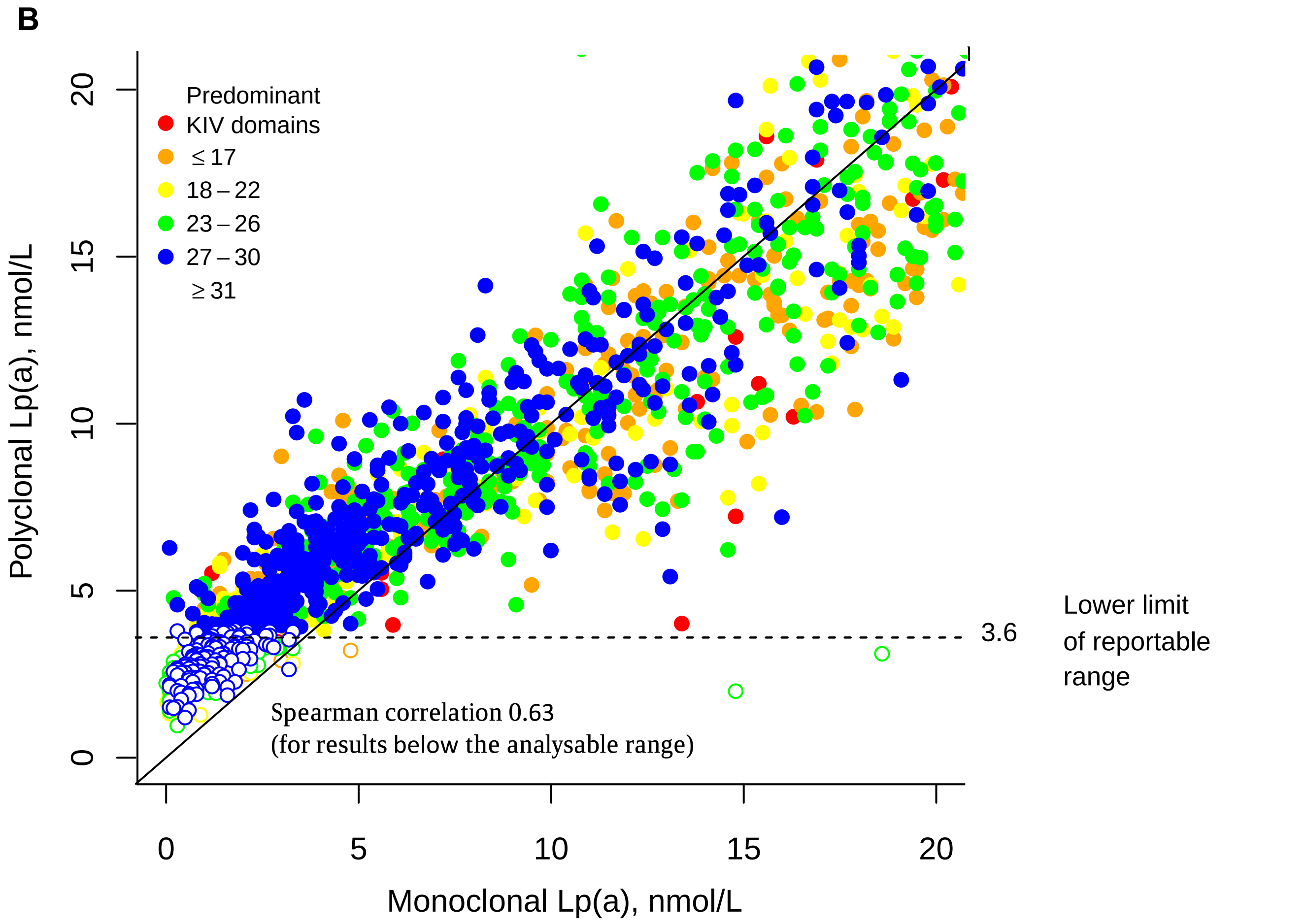

Supplemental Figure 1: Comparison of Lp(a) levels measured by the polyclonal and the (reference) monoclonal assays in 2967 one year samples: A) full range; B) magnified scale to show results below the lower limit of the reportable range.

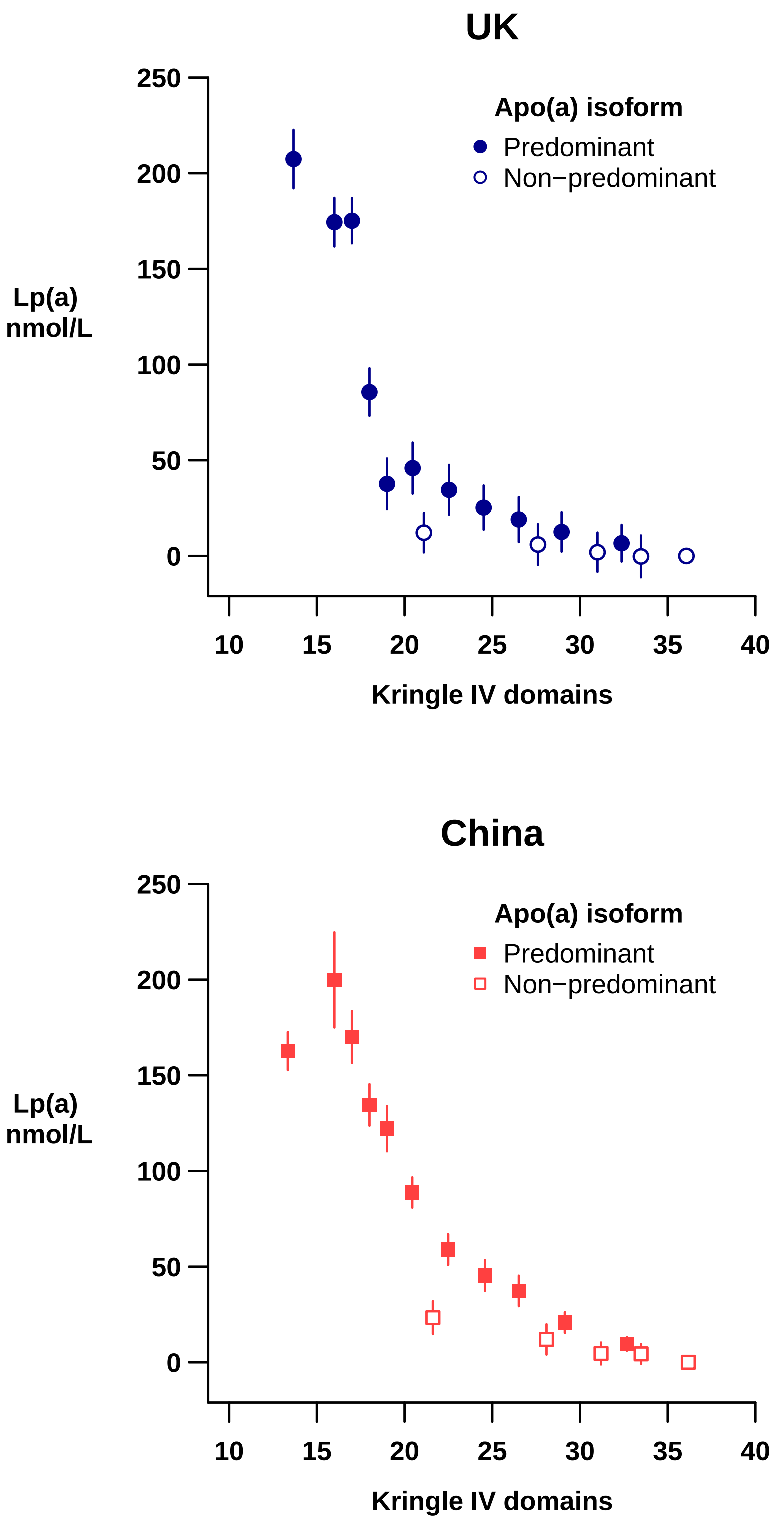

Supplemental Figure 2: Baseline Lp(a) levels associated with the number of kringle IV domains in the predominantly and non−predominantly expressed apo(a) isoforms jointly, in the UK and China. For the 5% of participants with only one isoform distinguishable,this was assumed to be both the predominant and the non−predominant isoform.

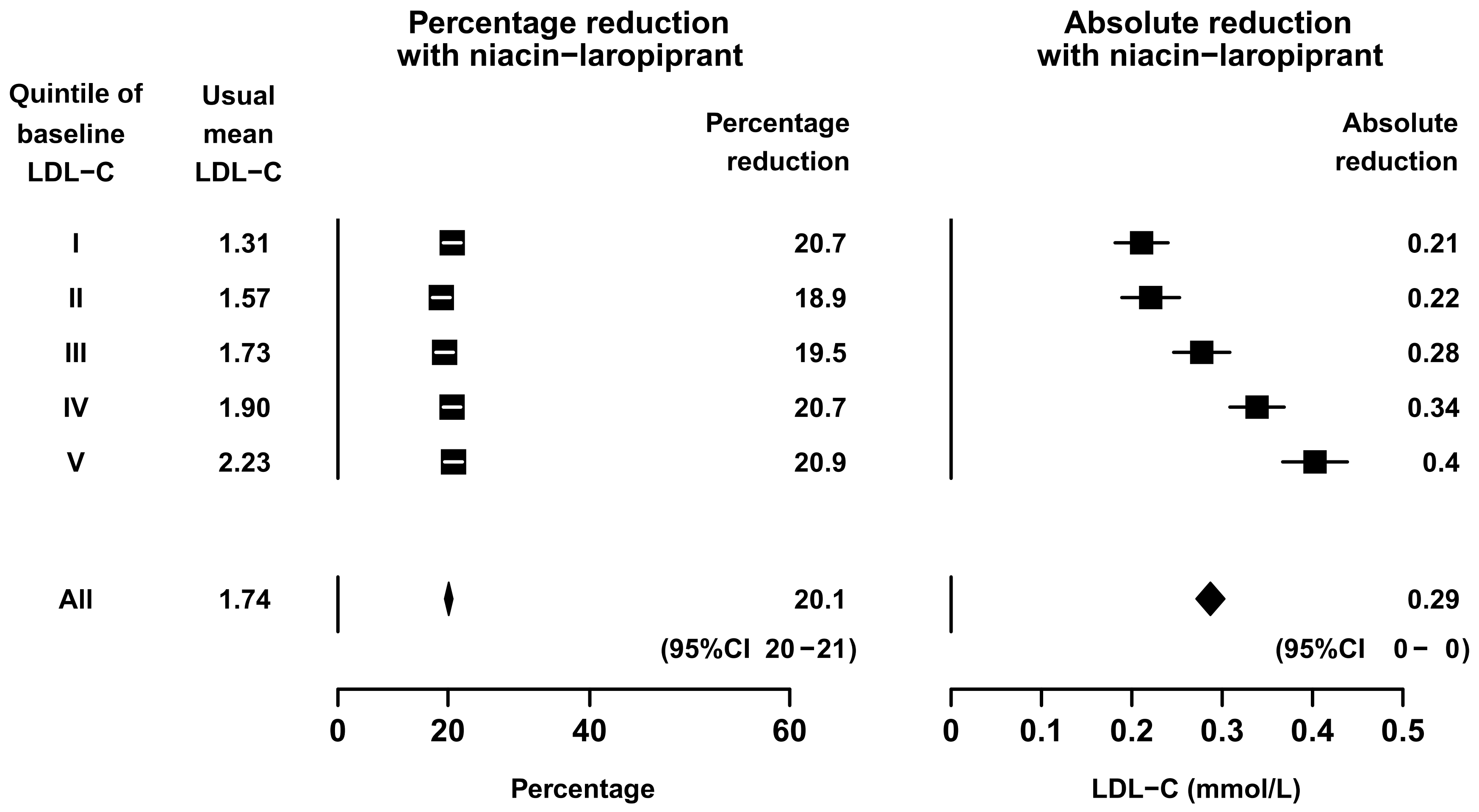

Supplemental Figure 3: Percentage and absolute reductions in LDL–C with niacin-laropiprant by fifths of baseline LDL-C
Percentage reduction panel shows adjusted LDL–C reductions based on modelling log LDL–C (see methods) and is plotted with an x–scale linear in the log LDL–C difference. Absolute reductions (with adjustment by stratification) are plotted on a linear scale. Usual mean LDL–C is the mean LDL–C in the placebo arm at 1 year.
